# Genetic Reagents for Making Split-GAL4 Lines in *Drosophila*

**DOI:** 10.1534/genetics.118.300682

**Published:** 2018-03-13

**Authors:** Heather Dionne, Karen L. Hibbard, Amanda Cavallaro, Jui-Chun Kao, Gerald M. Rubin

**Affiliations:** Janelia Research Campus, Howard Hughes Medical Institute, Ashburn, Virginia 20147

**Keywords:** gene expression, enhancer, genetic intersection, neuron, cell type

## Abstract

The ability to reproducibly target expression of transgenes to small, defined subsets of cells is a key experimental tool for understanding many biological processes. The *Drosophila* nervous system contains thousands of distinct cell types and it has generally not been possible to limit expression to one or a few cell types when using a single segment of genomic DNA as an enhancer to drive expression. Intersectional methods, in which expression of the transgene only occurs where two different enhancers overlap in their expression patterns, can be used to achieve the desired specificity. This report describes a set of over 2800 transgenic lines for use with the split-GAL4 intersectional method.

WE previously reported the expression patterns of over 7000 GAL4 lines in the adult nervous system of *Drosophila melanogaster* (Pfeiffer *et al.* 2008; Jenett *et al.* 2012; www.janelia.org/gal4-gen1). In these lines (referred to below as generation 1 GAL4 lines), expression of the transcription factor GAL4 is driven by a 2–3-kb segment of genomic DNA that contains one or more transcriptional enhancer sequences. While many lines show expression in a small fraction (< 1%) of neurons in the adult brain, only ∼1 in 1000 appears to drive expression in a single cell type (Jenett *et al.* 2012). To achieve greater specificity, we turned to the split-GAL4 intersectional method (Luan *et al.* 2006) using the optimized vectors described in Pfeiffer *et al.* (2010). In this method, individual enhancers drive the expression of either GAL4’s DNA-binding domain (DBD) or an activation domain (AD) joined to a leucine zipper-dimerization domain. When expressed individually, each half is insufficient to activate transcription of an upstream activating sequence (UAS) reporter. When both the AD and DBD are present in the same cell, they combine to make a functional transcription factor that can bind to tandem arrays of GAL4’s cognate UAS DNA sequence and activate transcription.

Here, we describe a set of such transgenic “hemidriver” lines expressing either the p65 AD or GAL4 DBD domain under the control of an enhancer from the collection described in Jenett *et al.* (2012). These lines have been used successfully to make comprehensive sets of lines that each show expression in one or a few cell types in a particular area of the brain; for example, the lamina of the optic lobe (Tuthill *et al.* 2013), mushroom body intrinsic and extrinsic neurons (Aso *et al.* 2014), and lobula columnar neurons (Wu *et al.* 2016). We discuss a sample screening protocol and the typical results that we obtain as a practical guide for those who want to use the hemidriver lines to develop split-GAL4 lines specific for additional cell types.

## Materials and Methods

### Construction of hemidriver lines

Lines were constructed essentially as described by Pfeiffer *et al.* (2010) using entry clones generated as described in Pfeiffer *et al.* (2008). To transfer the enhancer regions to split-GAL4 destination vectors, ∼50 ng of each entry clone was used in Gateway reactions with LR clonase (Thermo Fisher) and either pBPZpGAL4DBDUw or pBPp65ADZpUw (Pfeiffer *et al.* 2010; available from Addgene, plasmids 26233 and 26234, respectively). The p65 AD replaces the native GAL4 AD in pBPp65ADZpUw, which results in stronger transcriptional activation and insensitivity to inactivation by GAL80. BPp65ADZp and BPZpGDBD, hemidriver constructs that lack an enhancer fragment, were constructed by substituting the GAL4 coding sequence in pBPGAL4U (Pfeiffer *et al.* 2008) with the split-GAL4 coding sequences from pBPp65ADZpUw and pBPZpGAL4DBDUw, respectively, using *Kpn*I and *Hin*dIII sites. DNA for injections was prepared from 5-ml overnight cultures using a QIAprep kit (QIAGEN, Valencia, CA) and verified by *Eco*RI restriction digestion. The HL9 (Ddc), Tdc2 (Tyrosine decarboxylase 2), TH (ple), and Trh (Tryptophan hydroxylase) hemidrivers are described in Aso *et al.* (2014).

The DBD hemidrivers were inserted using φC31 site-specific integrase into the attP2 (3L) landing site (Groth *et al.* 2004) and the AD hemidrivers were inserted into the attP40 (2L) landing site (Markstein *et al.* 2008). The injections to generate the transformants were performed by Genetic Services (Cambridge, MA). Newly generated transformants were processed through a series of genetic crosses to remove the integrase source and to establish a homozygous stock, as diagrammed in Figure S1 of Pfeiffer *et al.* (2008). A subset of the DBD hemidrivers were balanced to facilitate further stock construction and efficient intersectional screening using the stock pJFRC200-10XUAS-IVS-myr::smGFP-HA in attP18, pJFRC216-13XLexAop2-IVS-myr::smGFP-V5 in su(Hw)attP8; wg*^Sp-1^/CyO*; *TM2/TM6B* (Nern *et al.* 2015). Some of the AD hemidrivers were balanced by a similar cross scheme utilizing one of the following stocks: *w*; *Amos^Tft^/CyO*; +, *w*; *wg^Sp-1^/CyO*; *MKRS/TM6B*, or *w*; *Kr^If-1^/CyO*,*2xTb-RFP*; *MKRS/TM6B*. Approximately 90% of the hemidriver lines could be maintained as homozygous viable stocks. Those lines that were homozygous lethal, sterile, or sickly were maintained over a balancer. We believe that in most or all cases, the lethality or sterility was due to background mutations in the stocks. We backcrossed ∼100 lethal or sterile AD lines for three generations to a *w^1118^* stock and were able to establish homozygous stocks in 90% of these cases.

### Data availability

The hemidriver lines shown in Supplemental Material, Table S1 have been deposited in the Bloomington *Drosophila* Stock Center (http://flystocks.bio.indiana.edu). The expression patterns driven by the parent generation 1 GAL4 and lexA lines are available at www.janelia.org/gal4-gen1. Supplemental material available at Figshare: https://doi.org/10.25386/genetics.5987542.

## Results and Discussion

### Using hemidriver lines to generate split-GAL4 lines with desired expression patterns

**Figure 1:**
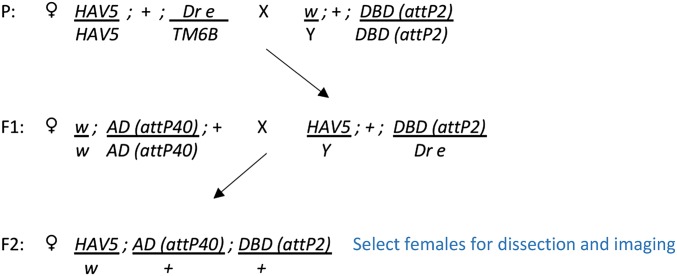
Crossing scheme used to generate split-GAL4 lines for screening. AD, activation domain; DBD, DNA-binding domain; P, parent; HAV, pJFRC200-10XUAS-IVS-myr::smGFP-HA (attP18), pJFRC216-13XLexAop2-IVS-myr::smGFP-V5 (su(Hw)attP8.

The first step in generating a split-GAL4 line for a given cell type is screening the images of generation 1 GAL4 lines (Jenett *et al.* 2012; www.janelia.org/gal4-gen1) to identify enhancers that drive expression in the desired cell type. Once a list of enhancers has been identified, AD and DBD hemidrivers that use those enhancers can be selected from the lines in Table S1 and crossed using a cross scheme such as that shown in Figure 1.

**Figure 2:**
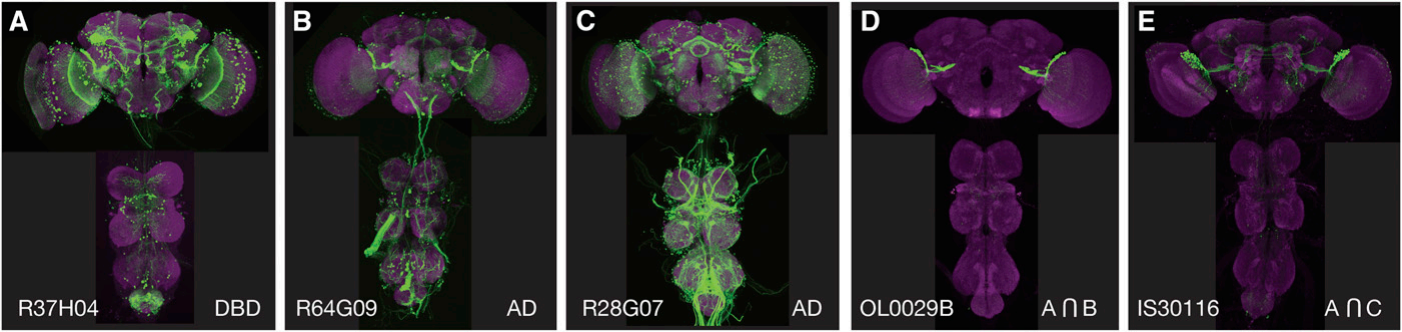
Two intersections attempting to make a split-GAL4 driver line specific for the LPLC1 cell type. (A–C) Expression patterns of the generation 1 GAL4 lines R37H04 (A), R64G09 (B), and R28G07 (C) observed with insertion of the transgene in attP2 (Jenett *et al.* 2012). (D) The result of an intersection between the R37H04-DBD (in attP2) and R64G09-AD (in attP40) lines; highly specific expression in the desired cell type is observed. (E) The result of an intersection between the R37H04-DBD (in attP2) and R28G07-AD (in attP40) lines; expression in the desired cell type is observed, but now with off-target expression in other cells. AD, activation domain; DBD, DNA-binding domain.

The F2 generation of potentially useful AD and DBD hemidriver combinations is then screened for GAL4-driven expression. Based on work done in several laboratories at Janelia, where ∼20,000 such crosses have been examined, between 5 and 30% of the intersections show expression in a restricted set of cells. However, no more than 5% of the attempted intersections generated flies that lacked detectable expression in off-target cells and could be considered specific for the target cell type. Figure 2 shows two intersections aimed at producing a driver line specific for the visual projection neuron LCLP1 (Wu *et al.* 2016). The same DBD line was used in both crosses, but in combination with different AD lines. Both crosses yielded the desired cell type, but only in one case was it free from expression in off-target cells.

How much off-target expression is acceptable depends on the intended use of the lines. A few off-target cells may not be a problem if the intended use of the split-GAL4 line is anatomical analysis, functional imaging, or as a guide for the placement of a recording electrode. On the other hand, for behavioral screens involving the activation of cell types, and to a lesser extent inactivation of cell types, off-target expression can be confounding; such off-target expression can also occur in nonneuronal cells such as muscle, which will not be observed if only dissected nervous systems are examined [see Aso *et al.* (2014)]. Having multiple independent intersections for a given cell type can be helpful here, as these lines will almost always have different off-target expression. Use of multiple split-GAL4 lines for a cell type that were derived using different hemidriver lines also decreases the chance of being misled by effects of genetic background; while the general background of all the lines is the same there may be mutations associated with the chromosomes carrying the individual AD or DBD transgenes. These could affect fly behavior but are less likely to be the same for multiple AD/DBD combinations. Making the lines more specific by “triple intersections” (see for example, Dolan *et al.* 2017) can also be attempted. Finally, it is important to keep in mind that the expression pattern observed with any GAL4 driver depends on the chromosomal location and number of UASs carried by the indicator gene [see Pfeiffer *et al.* (2010) and Aso *et al.* (2014) for examples]. For this reason, it is best to use indicator and effector lines inserted at the same site; in the ideal case, both the indicator and effector functions are provided by the same gene product, for example, 20XUAS-IVS-CsChrimson-mVenus (Klapoetke *et al.* 2014).

Screening is rapid enough that this has generally not been a limiting factor; however, several factors significantly influence the screening success rate:

1. The degree of certainty with which the selected enhancers can be said to express in the desired cell type is perhaps the largest factor affecting success. Many generation 1 GAL4 lines express in patterns that are too broad to allow the identification of individual cell types with confidence and different cell types may have indistinguishable morphologies at this level of imaging resolution. In some cases, we have found it helpful to use stochastic labeling with the Multi-Color-Flip-Out method (Nern *et al.* 2015) to visualize the morphologies of individual cells in generation 1 lines as a way of discerning what cell types are present.
2. Because expression patterns vary with insertion site, the hemidriver expression pattern may differ from that of the generation 1 GAL4 line with the same enhancer because the AD hemidrivers are inserted into attP40 and the generation 1 lines are inserted in attP2. Different insertion sites for the AD and DBD hemidrivers were used to avoid transvection (Mellert and Truman 2012) and to make it possible to generate stable split-GAL4 lines with both an AD and DBD hemidriver. For many of the enhancers used in Table S1, enhancer-LexA constructs inserted in attP40 have been generated and imaged (www.janelia.org/gal4-gen1), and these images can serve as a useful guide for the expression pattern of the enhancer inserted at that landing site. Examples of three such comparisons are shown in Figure 3. In cases where the expression pattern is not reproduced in attP40 and the number of enhancers for the desired cell type is limited, we have had success by reinjecting the AD hemidriver into additional landing sites that were selected to be more similar to attP2, such as JK22C and JK73A (Knapp *et al.* 2015). Since the DBD hemidrivers are inserted into the same site as the generation 1 GAL4 lines, they reliably reproduce the GAL4 expression pattern. For this reason, a useful strategy is to use an AD hemidriver that is empirically known to express in the target cell type and cross it to DBD hemidrivers from a set of lines selected based on anatomy.

**Figure 3:**
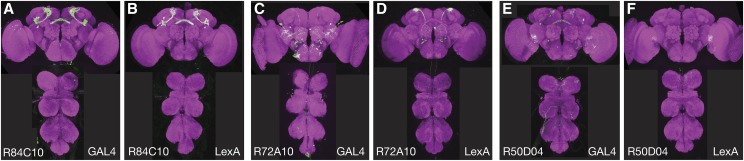
Comparison of the expression patterns driven by three enhancer fragments when inserted as GAL4 constructs in attP2 and as lexA constructs in attP40. (A and B) Enhancer R84C10 drives similar expression in both sites. (C and D) Enhancer R72A10 drives in a more restricted pattern in attP40, but the cell type indicated by the arrow is more prominent. (E and F) R50D04 has a more restricted pattern in attP40 and the cell type indicated by the arrow is not observed. All images are from Jenett *et al.* (2012) and confocal stacks can be downloaded from www.janelia.org/gal4-gen1.
3. A successful intersection requires the identification of at least two generation 1 lines that express in the cell type of interest; for some cell types, the lines in Table S1 will be insufficient for this purpose. In this case, there is little choice but to expand the set of hemidriver lines. For example, we have been able to obtain successful intersection in such cases by also incorporating the set of hemidrivers described in Tirian *et al.* (2017) that were generated in the same vectors but used a different set of enhancers. Also, additional hemidriver constructs can be generated if an enhancer is identified for which a hemidriver line does not already exist in either collection. We note that the set of hemidriver lines in Table S1 uses less than half the enhancers described in Jenett *et al.* (2012) and that the selection of these enhancers was biased by the anatomical interests of a small number of laboratories. The hemidrivers in these collections can also be used in combinations with hemidrivers generated by other strategies (Gohl *et al.* 2011; Diao *et al.* 2015; Simpson 2016); for example, Aso *et al.* (2014) used a hemidriver made using a large chromosomal region of the tyrosine hydroxylase gene to make split-GAL4 lines for subsets of dopaminergic neurons.

**Figure 4:**
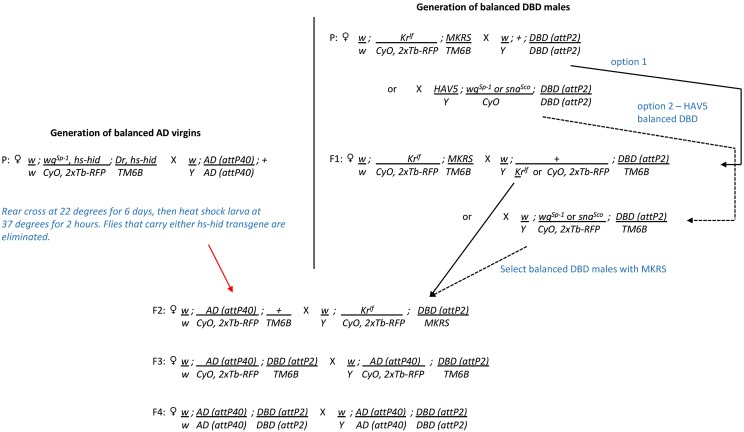
Crossing scheme used to construct homozygous stable split-GAL4 lines. AD, activation domain; DBD, DNA-binding domain; P, parent.

Once combinations of hemidrivers that yield successful intersections have been identified in the screening process, we generally construct stable split-GAL4 lines that carry a combination of the two hemidrivers, using the cross scheme shown in Figure 4. In cases where uniformity of genetic background is of paramount importance, the hemidrivers can be backcrossed to a common stock before making stable lines. This is usually not an issue; however, as in the general use case, the split-GAL4 line’s chromosomes are heterozygous with those of a common indicator or effector line in the experimental flies that are being assayed.

### Conclusions

Using the combined hemidriver collections described in this paper and Tirian *et al.* (2017), our experience in several brain areas suggests that it will be possible to obtain useful split-GAL4 lines for more than three-quarters of the cell types in the adult fly brain. By making additional hemidrivers this percentage could likely be increased. However, we have also found cases where it has not been possible to make split-GAL4 lines that separate two closely related, but clearly morphologically distinct, cell types. This may be a fundamental limitation based on the biology of combinatorial gene control; there may simply be no two enhancers that uniquely share an intersection in those individual cell types.

We anticipate rapid progress on electron microscopic level connectomics in *Drosophila* [for example, Eichler *et al.* (2017), Takemura *et al.* (2017), and Zheng *et al.* (2017)]. These efforts will produce the morphologies of all the neurons participating in particular circuits. Such cell shapes can serve as templates for searching for enhancers that drive expression in the same cell types. These enhancers can then be used to generate the split-GAL4 drivers needed to manipulate those cells or image their activity. Clean drivers, as can be generated by these methods, will permit the manipulation of specific cell types in freely moving flies. Generating these lines will be, of necessity, a community effort, accomplished most efficiently by using shared reagents such as those described here.
